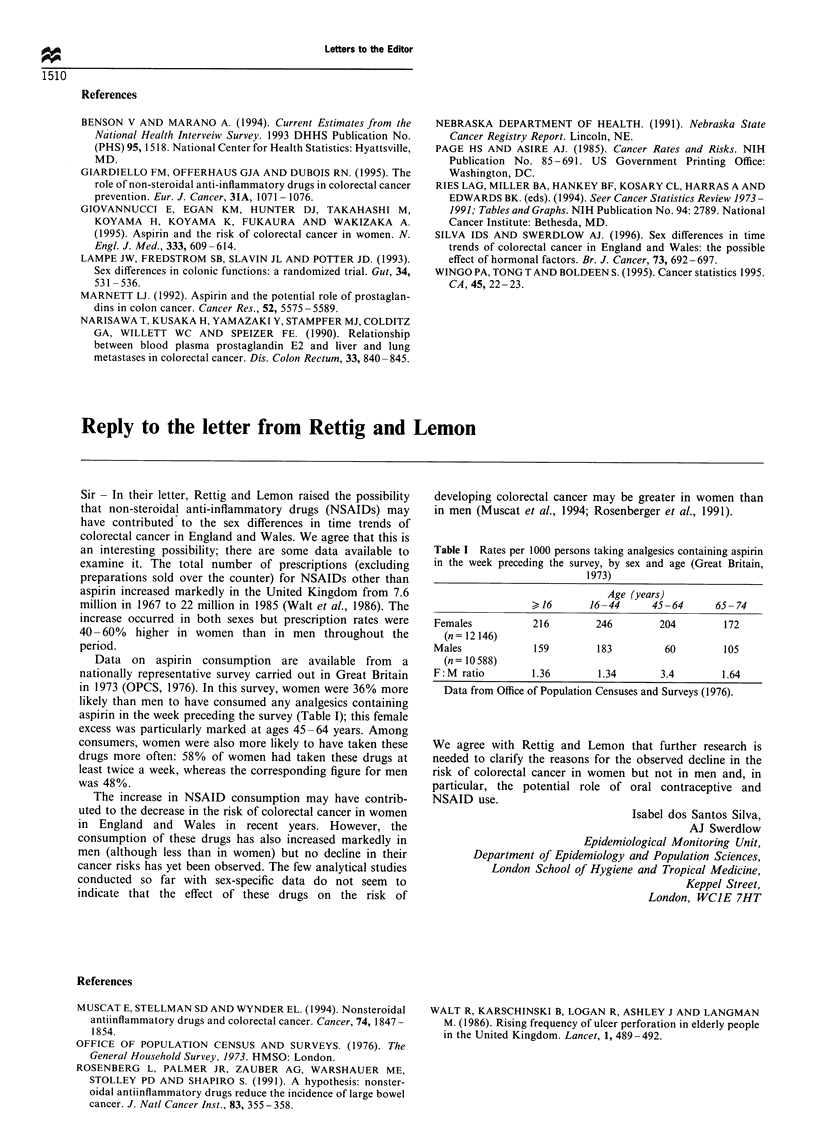# Reply to the letter from Rettig and Lemon

**Published:** 1996-11

**Authors:** Isabel dos Santos Silva, AJ Swerdlow


### Reply to the letter from Rettig and Lemon

Sir – In their letter, Rettig and Lemon raised the possibility that non-steroidal anti-inflammatory drugs (NSAIDs) may have contributed to the sex differences in time trends of colorectal cancer in England and Wales. We agree that this is an interesting possibility; there are some data available to examine it. The total number of prescriptions (excluding preparations sold over the counter) for NSAIDs other than aspirin increased markedly in the United Kingdom from 7.6 million in 1967 to 22 million in 1985 (Walt *et al.,* 1986). The increase occurred in both sexes but prescription rates were 40–60% higher in women than in men throughout the period.

Data on aspirin consumption are available from a nationally representative survey carried out in Great Britain in 1973 (OPCS, 1976). In this survey, women were 36% more likely than men to have consumed any analgesics containing aspirin in the week preceding the survey (Table I); this female excess was particularly marked at ages 45–64 years. Among consumers, women were also more likely to have taken these drugs more often: 58% of women had taken these drugs at least twice a week, whereas the corresponding figure for men was 48%.

The increase in NSAID consumption may have contributed to the decrease in the risk of colorectal cancer in women in England and Wales in recent years. However, the consumption of these drugs has also increased markedly in men (although less than in women) but no decline in their cancer risks has yet been observed. The few analytical studies conducted so far with sex-specific data do not seem to indicate that the effect of these drugs on the risk of developing colorectal cancer may be greater in women than in men (Muscat *et al.,* 1994; Rosenberger *et al.,* 1991).

**Table I** Rates per 1000 persons taking analgesics containing aspirin in the week preceding the survey, by sex and age (Great Britain, 1973)

| | *Age (years)* | | | |
|---|---|---|---|---|
| | *≥16* | *16–44* | *45–64* | *65–74* |
| Females ($n$ = 12 146) | 216 | 246 | 204 | 172 |
| Males ($n$ = 10 588) | 159 | 183 | 60 | 105 |
| F:M ratio | 1.36 | 1.34 | 3.4 | 1.64 |

Data from Office of Population Censuses and Surveys (1976).

We agree with Rettig and Lemon that further research is needed to clarify the reasons for the observed decline in the risk of colorectal cancer in women but not in men and, in particular, the potential role of oral contraceptive and NSAID use.

Isabel dos Santos Silva,
AJ Swerdlow
*Epidemiological Monitoring Unit,*
*Department of Epidemiology and Population Sciences,*
*London School of Hygiene and Tropical Medicine,*
*Keppel Street,*
*London, WC1E 7HT*

**References**

MUSCAT E, STELLMAN SD AND WYNDER EL. (1994). Nonsteroidal antiinflammatory drugs and colorectal cancer. *Cancer,* **74,** 1847–1854.
OFFICE OF POPULATION CENSUS AND SURVEYS. (1976). *The General Household Survey, 1973.* HMSO: London.
ROSENBERG L, PALMER JR, ZAUBER AG, WARSHAUER ME, STOLLEY PD AND SHAPIRO S. (1991). A hypothesis: nonsteroidal antiinflammatory drugs reduce the incidence of large bowel cancer. *J. Natl Cancer Inst.,* **83,** 355–358.
WALT R, KARSCHINSKI B, LOGAN R, ASHLEY J AND LANGMAN M. (1986). Rising frequency of ulcer perforation in elderly people in the United Kingdom. *Lancet,* **1,** 489–492.